# Treatment with epigenetic agents profoundly inhibits tumor growth in leiomyosarcoma

**DOI:** 10.18632/oncotarget.25056

**Published:** 2018-04-10

**Authors:** Cynthia De Carvalho Fischer, Yue Hu, Michael Morreale, Wan Ying Lin, Akhil Wali, Maya Thakar, Enusha Karunasena, Rupashree Sen, Yi Cai, Lauren Murphy, Cynthia A. Zahnow, Harold Keer, Manjusha Thakar, Nita Ahuja

**Affiliations:** ^1^ Department of Surgery, Johns Hopkins University School of Medicine, Baltimore, MD, United States; ^2^ Institut für Allgemein, Viszeral und Transplantationschirurgie, Charite Universitätsmedizin Berlin, Berlin, Germany; ^3^ Department of Surgical Oncology, The Second Affiliated Hospital of Zhejiang University, School of Medicine, Hangzhou, Zhejiang, P.R. China; ^4^ Department of Oncology, Johns Hopkins University School of Medicine, Baltimore, MD, United States; ^5^ Astex Pharmaceuticals Inc., Pleasanton, CA, United States; ^6^ Department of Urology, Johns Hopkins University School of Medicine, Baltimore, MD, United States

**Keywords:** leiomyosarcoma, 5-azacitidine, Guadecitabine

## Abstract

Leiomyosarcomas are rare mesenchymal neoplasms characterized by a smooth muscle differentiation pattern. Due to the extremely poor prognosis in patients, the development of novel chemotherapeutic regimens remains critically important. In this study, multiple leiomyosarcoma cell lines, SK-UT1, SK-LMS1, and MES-SA were treated with varying doses of the DNA Methyltransferase Inhibitors (DNMTi) 5-azacitidine (Aza), 5-aza-2-deoxycytidine (DAC), and guadecitabine (SGI-110). The effect of these epigenetic modulators was measured using both *in-vitro* and *in-vivo* models.

Of the three epigenetic modulators, Guadecitabine was the most effective at decreasing cell survival in LMS cell lines. SK-UT1 was found to be the more sensitive to all three epigenetic modulators, while SK-LMS1 and MES-SA were more resistant. The contrast in sensitivity seen was also represented by the increase in apoptosis in Aza and guadecitabine. In parallel with Aza, guadecitabine was observed to also arrest the cell cycle.

Treatment with guadecitabine led to a decrease in growth across the spectrum of sensitivity in LMS cell lines, both in a delayed *in vitro* and *in vivo* model; in parallel experiments, apoptotic pathways were activated in sensitive and less sensitive lines. Additional studies are required to explore potential therapeutic applications and mechanisms for leiomyosarcoma treatment.

## INTRODUCTION

Sarcomas are rare tumours that arise from mesenchymal tissues and comprise 1% of all adult solid cancers [1]. Leiomyosarcomas (LMS) are sarcomas that comprise spindle-cell neoplasms that develop from smooth muscle tissue and affect approximately 1 out of 100,000 Americans per year [2–4].

Though LMS may arise from any site in the body containing a smooth muscle layer, they most often occur in the uterus, the retroperitoneum, and the extremities [5, 6]. Although a detailed characterization of the biological and clinical characteristics of this form of cancer is lacking, patients with LMS have a poor prognosis, high recurrence rates, and a minimal response to standard chemotherapeutic treatment. A retrospective study carried out by Kapp *et al.* revealed that the overall five-year disease specific survival rates for stage III and stage IV disease were 44.9% and 27.7% respectively [7].

The exact pathophysiology of LMS is poorly understood. Recent genomic studies in LMS have revealed high chromosomal instability and identified mutations in driver genes that lead to the activation of cell proliferation signaling and anti-apoptotic pathways [8, 9]. For example, an analysis of The Cancer Genome Atlas (TCGA) data belonging to 98 primary LMS revealed frequent mutations of driver genes such as TP53, RB1, and ATRX1 [10]. A similar study conducted using the COSMIC database of 107 primary LMS revealed similar results, in that TP53 and ATRX were both mutated in 23% of cases, and MED12 in 8% [11]. Recently published genomic and epigenomic data by the TCGA on multiple sarcomas, including LMS, compared with other gynecologic and soft tissue sarcomas demonstrates a unique methylation patterns distinct to LMS typically showing hypomethylation compared with other sarcomas. Notably in our data cell line SK-LMS1 was previously described as being hypomethylated compared with the cell line SK-UT1, and as noted by the TCGA, several sarcoma types (including some LMS) show patterns of methylation that are unique and not representative of a histology. Thus, further supporting the results of this study that individual LMS may respond to epigenetic therapies differently based on their epi-phenotype [12].

Furthermore, retrospective studies have indicated that high expression of molecules such as BCL2 correlates with poor prognosis [13]. Similarly, p16 has been implicated in tumorigenesis as well. A comparison study carried out showed that the hyper-expression of p16 in smooth muscle uterine tumor patients diagnosed with LMS played an important role in sarcomagenesis [14]. On the other hand, a second study has shown that hyper-methylation, leading to a loss of p16 expression, correlates with significant increases in tumor size in soft tissue LMS patients [15].

Epigenetic alterations in LMS have not been extensively studied so far. The benefits of sarcoma epigenetics are that it focuses on modifications to heritable genomic variations, which do not affect the genetic code. These alterations may lead to changes in various cellular processes as well as the overall cellular phenotype. Epigenetic alterations such as changes in patterns of DNA-methylation and complex alterations in chromatin structure contribute to all stages of tumour development i.e. initiation, progression, proliferation and metastasis [16]. Hypermethylation of CpG islands in promotor regions often result in the transcriptional silencing of downstream genes, and has been shown to occur in most forms of cancer [17–19].

Epigenetic modulators such as 5-azacitidine (Aza) and 5-aza-2-deoxycytidine (DAC) are FDA-approved DNA Methyltransferase Inhibitors (DNMTi) that function as cytosine nucleoside analogues, and inhibitors of DNA-methyl transferases. Both Aza and DAC are clinically approved for treatment in patients with haematological malignancies such as myelodysplastic syndrome. Epigenetic modulators have also been employed as primary combination epigenetic therapy using DNMTi and Histone Deacetylase (HDAC) inhibitors in the context of clinical trials for solid neoplasms such as breast cancer, lung, and colorectal [20–24]. Furthermore, recent studies have shown promise using these agents to reverse chemoresistance [25, 26]. Success of treatment is dependent on prolonged administration of the drug as shown by Silverman *et al.*, who demonstrated that patients required several months of treatment in myelodysplastic syndromes prior to response, while other studies have shown that underlying gene mutations may increase sensitivity [27, 28]. In addition, these drugs have a short half-life, leading to interest in the development of stable, longer acting epigenetic drugs. Guadecitabine (SGI-110) is a novel, small-molecular DNMTi agent that couples both DAC and deoxyguanosine, whose resistance to cytidine deaminase has been shown to lead to a longer half-life in an aqueous solution [29]. The gradual cleavage of the guadecitabine into decitabine leads to a more pro-longed, stable release of the drug, as opposed to DAC’s short-term peak in plasma concentration. This has been hypothesized to increase the efficacy of guadecitabine treatment in advanced stage diseases such as acute myeloid leukaemia [30]. In pre-clinical studies, guadecitabine reverses chemotherapy resistance to cisplatin in multiple ovarian cancer cell lines *in vitro*, in addition to inhibiting tumor growth *in vivo* [29]. A Phase 1 clinical trial in myelodysplastic syndrome and acute myeloid leukaemia demonstrated sustained demethylation of repetitive elements such as LINE1 in the genome [31].

We assessed the anti-proliferative and pro-apoptotic effects of epigenetic modulators such as Aza, DAC, and guadecitabine on the growth of leiomyosarcoma cells, employing both *in vitro* and *in vivo* models. Previously, there has been little focus on the epigenetic landscape of this cancer, and the role of DNA-methylation in the etiology and progression of this cancer type remains mostly unexplored. Our goal was to identify whether the use of DNMTi’s have anti-proliferative or pro-apoptotic effects, as well as to explore how our investigational demethylating agent guadecitabine compared to previously established treatments such as Aza and DAC.

## RESULTS

### Leiomyosarcoma cell lines showed variable responses to epigenetic drugs *in vitro*

The cellular viability of three leiomyosarcoma cell lines (SKUT1, SK-LMS1, and MES-SA) was measured using an MTT assay following treatment with various concentrations of Aza, DAC and guadecitabine. Cell lines were treated with the various DNMTi’s for a variable number of days (range of treatment: 1–5 days) and then a MTT assay was performed to assess cell viability. Cell viability at each time point was calculated relative to the respective control and expressed as a percentage value.

**Figure 1:**
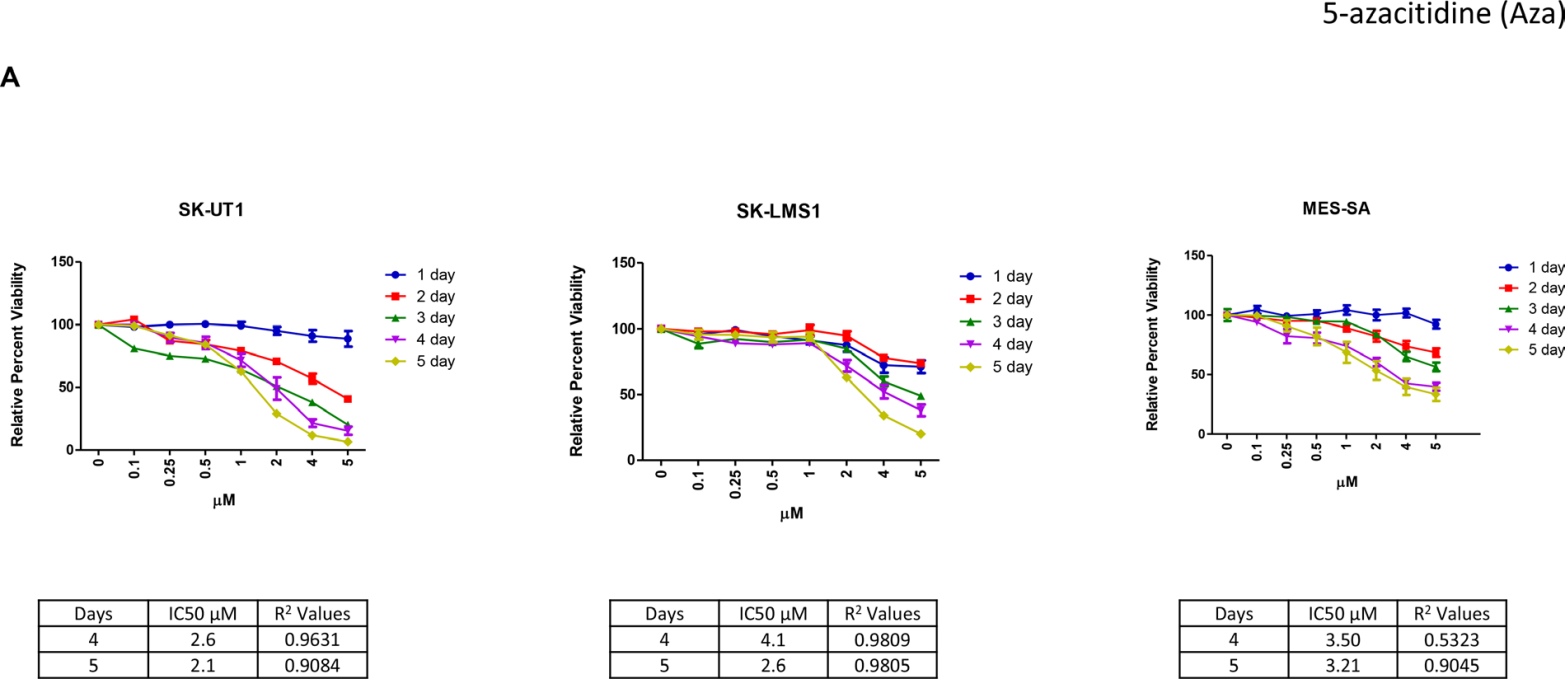

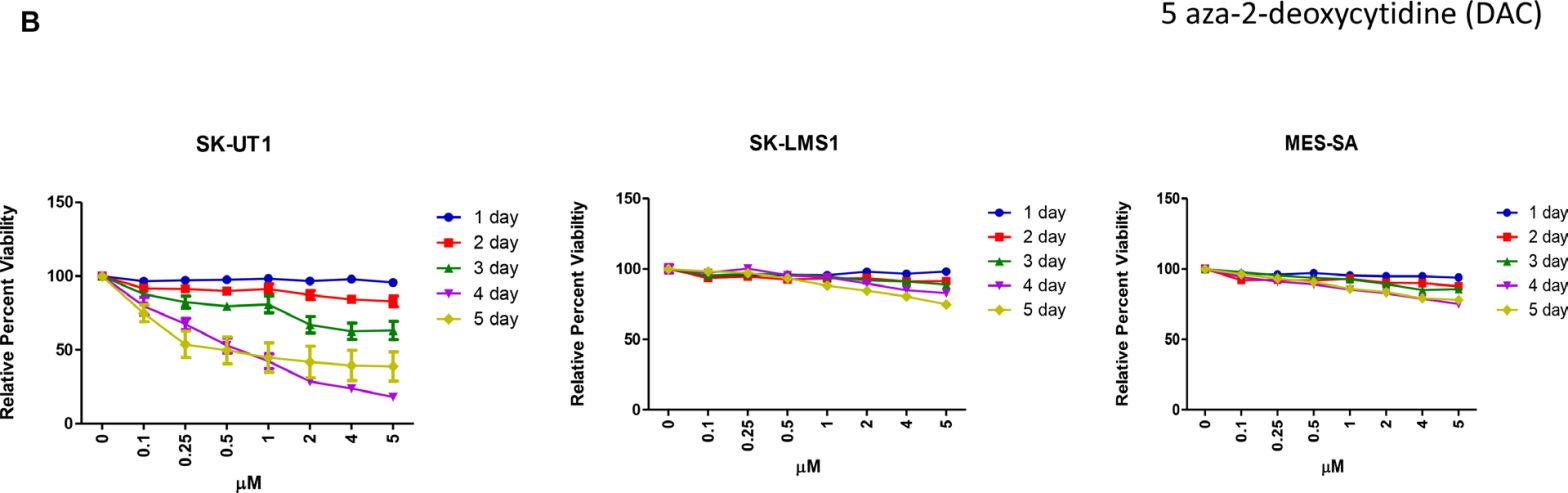

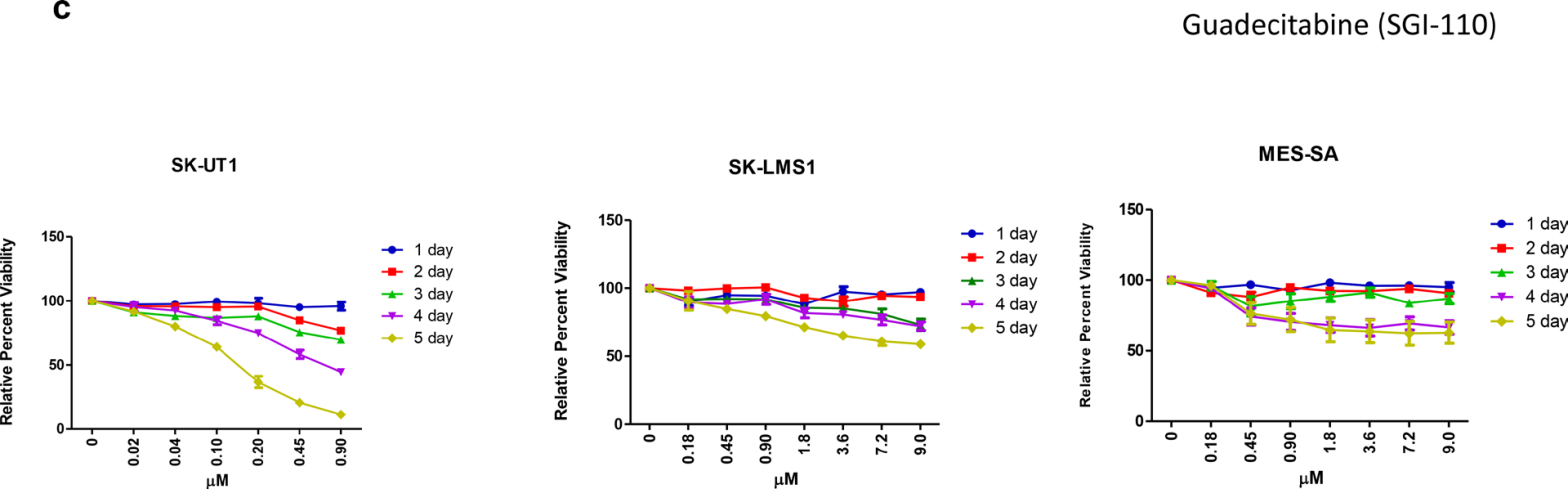

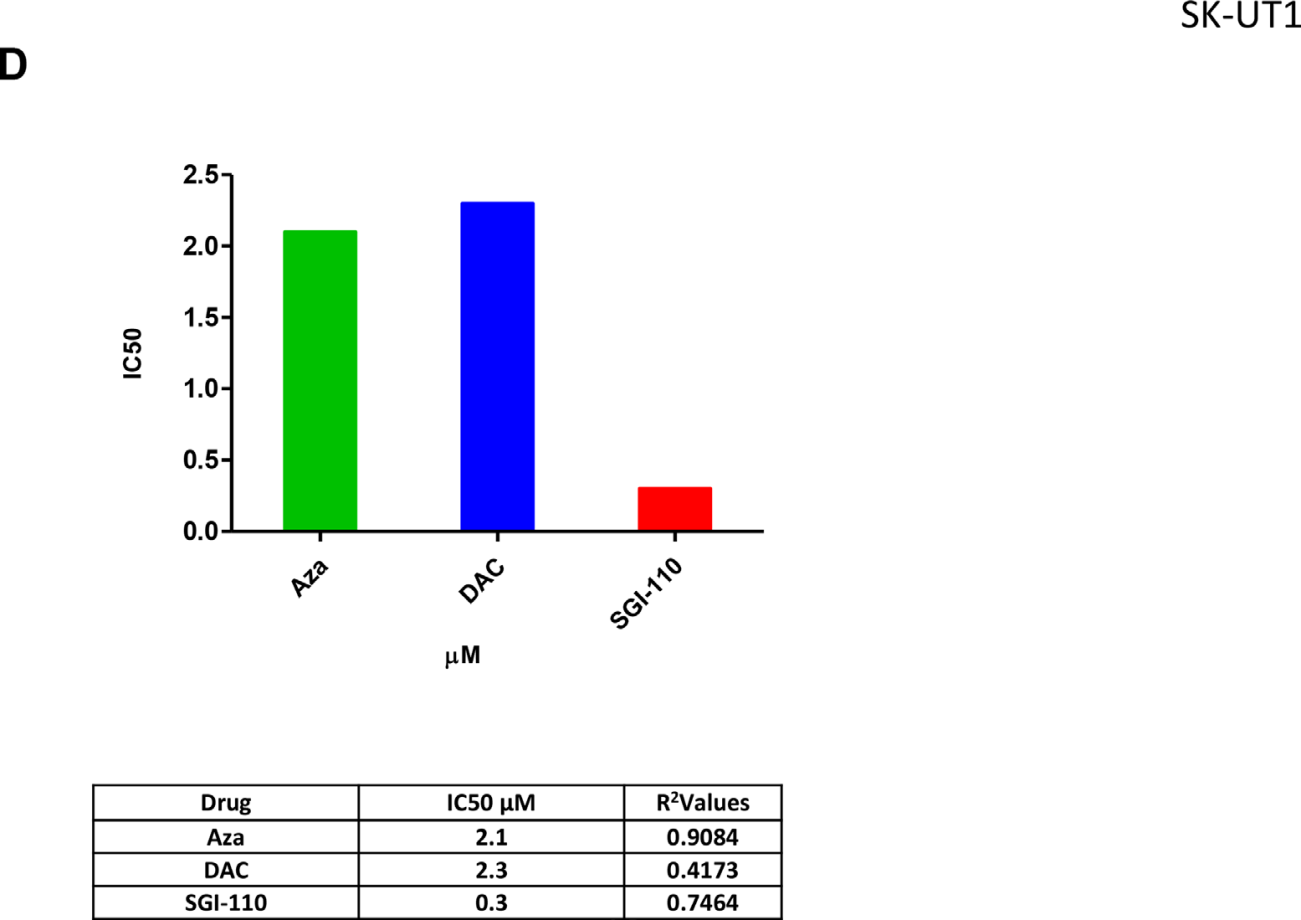
LMS cell lines showed variable responses to epigenetic modulators *in vitro* Cells were freshly treated with different concentrations of epigenetic modulators for 1–5 days (**A**, **B**, **C**). At the end of the incubation period, a standard MTT assay was carried out. The percent viability was calculated by comparing each data point to the control and the final value was expressed in percentage. The IC_50_ was calculated by using the slope equation, as mentioned in the methods. The obtainable IC_50_ values for all cell lines were compared for each epigenetic modulator and was plotted using Graph pad prism (**D**). Data shown represents mean ± SEM (1A–1C). Data shown represents mean (1D).

Overall, individual LMS cell lines showed differential sensitivity to each of the DNMTi’s. Amongst the different DNMTi drugs that were tested with SK-UT1, guadecitabine elicited the most potent anti-proliferative response; requiring concentrations ten-times lower than Aza and DAC to effectively inhibit cell growth. Based on how effective each of the DNMTi’s were at inhibiting cellular growth (Figure 1A–1C), SK-UT1 showed the highest sensitivity to each of the DNMTi’s while SK-LMS1 and MES-SA were less sensitive.

In order to further quantify anti-proliferative responses, an IC_50_-value was calculated as mentioned previously. After five days of treatment with DNMTi agents, SK-UT1 was the most sensitive cell line demonstrating an IC_50_ value of 2.1 μM for Aza, 2.3 μM for DAC and 0.3 μM for guadecitabine. MES-SA proved to be relatively un-sensitive to any of the three drugs. Only after 4 days (3.5 μM) and 5 days (3.21 μM) of treatment with Aza did it reach 50% viability. SK-LMS1 was equally non-responsive, again reaching 50% viability only after 4 and 5 days of treatment with Aza (with IC_50_ values of 4.1 μM and 2.6 μM, respectively). Since all three DNMTi’s reached 50% viability in the SK-UT1 cell line after 5 days of treatment, these conditions were used to determine which DNMTi was the most effective (Figure 1D). Guadecitabine had an IC_50_ almost ten times smaller than Aza or DAC, and Aza was slightly more effective than DAC. We next examined the mechanism of these epigenetic modulators further using Aza and guadecitabine, as they showed effects at the lowest doses.

### Treatment with epigenetic modulators leads to an increase in apoptosis through upregulation of Caspase 3/7 activity

**Figure 2:**
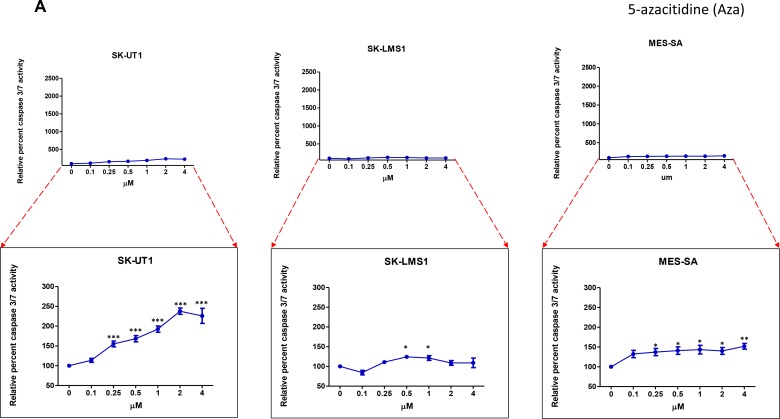

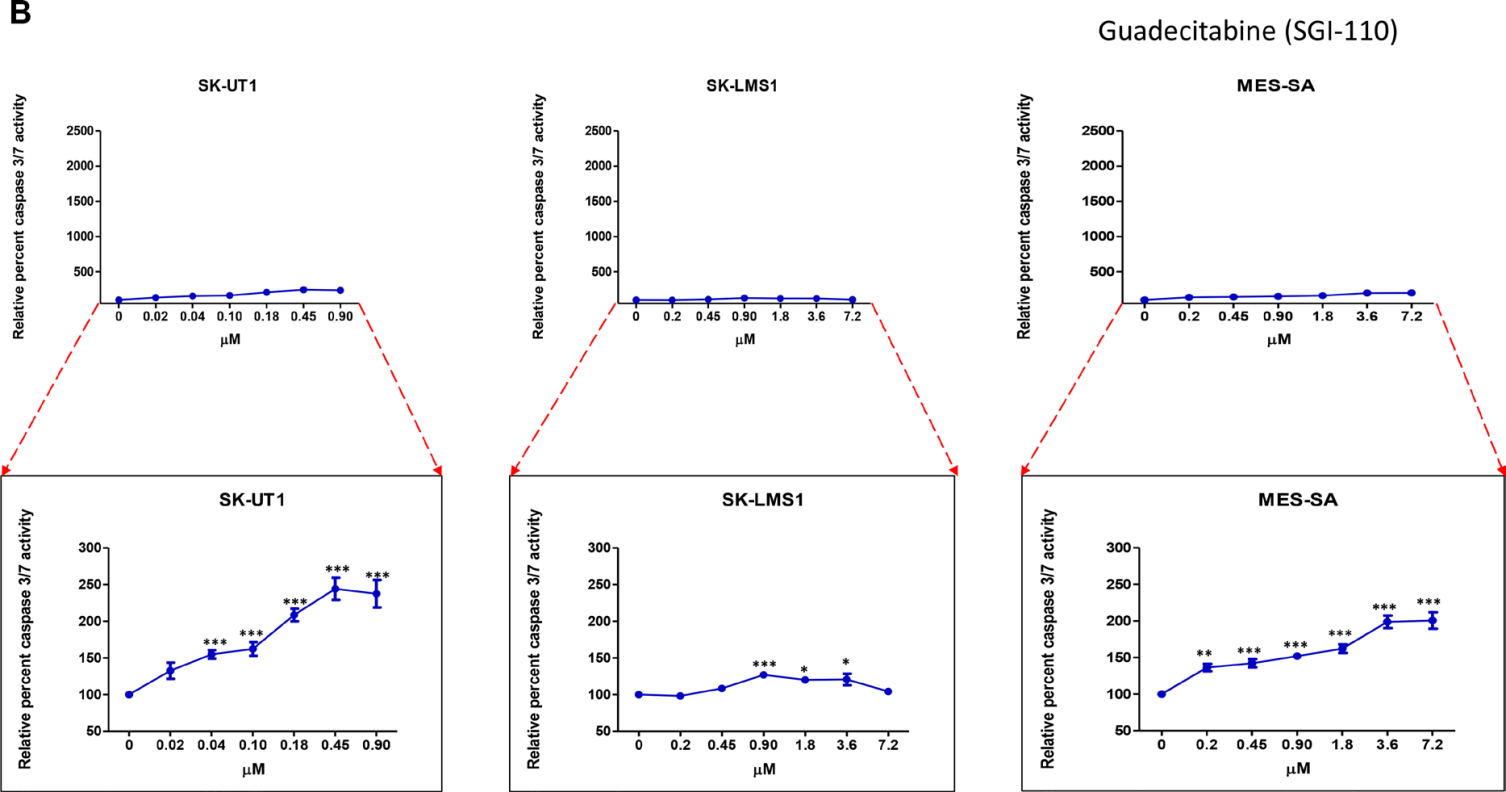
Up-regulation of Caspase 3/7 activity in presence of epigenetic modulators All three cell lines were treated for three days with varying concentrations of the Aza (**A**) or guadecitabine (**B**). The caspase 3/7 activity was measured using the luminescence kit discussed in Methods. Each data point was compared with the control; each value is plotted in terms of percentage. Statistical analysis was done using one-way ANOVA using Tukeys multiple comparison test wherein each point was compared with the control. Data shown represents mean ± SEM. Bottom graph represents a magnification along the Y axis of the top graph.

A Caspase 3/7 Glow Assay was used to detect Caspase 3 and 7 as surrogate markers of apoptotic activity in order to better understand how Aza and guadecitabine inhibit cellular viability immediately following three days of treatment (Figure 2A and 2B).

For SK-UT1 and MES-SA, we found that guadecitabine elicited higher caspase 3/7 activity when compared to Aza. For example, in the SK-UT1 cell line, guadecitabine increased caspase activity to by 50% of the untreated control at a drug concentration of 0.04 µM, while the same response was not seen with Aza until a dosage of 0.25 µM was reached. Guadecitabine, therefore, produced an increase in caspase levels at over six times a lower concentration than Aza. In MES-SA, treatment of guadecitabine at a dosage of 0.45 µM was enough to cause an increase in caspase activity by 50% of the controls, while Aza did not produce as profound of a caspase response comparable to the responses observed with guadecitabine. SK-LMS1, on the other hand, did not show any significant difference in caspase response between the two epigenetic modulators.

As per the above results, guadecitabine was the more effective DNMTi at inhibiting growth in cell lines with varying sensitivity, while also inducing apoptotic pathways. Consequently, we next tested these results on a three dimensional model using a clonogenic assay (colony formation assay).

### Guadecitabine inhibits colony formation in LMS cell lines

**Figure 3:**
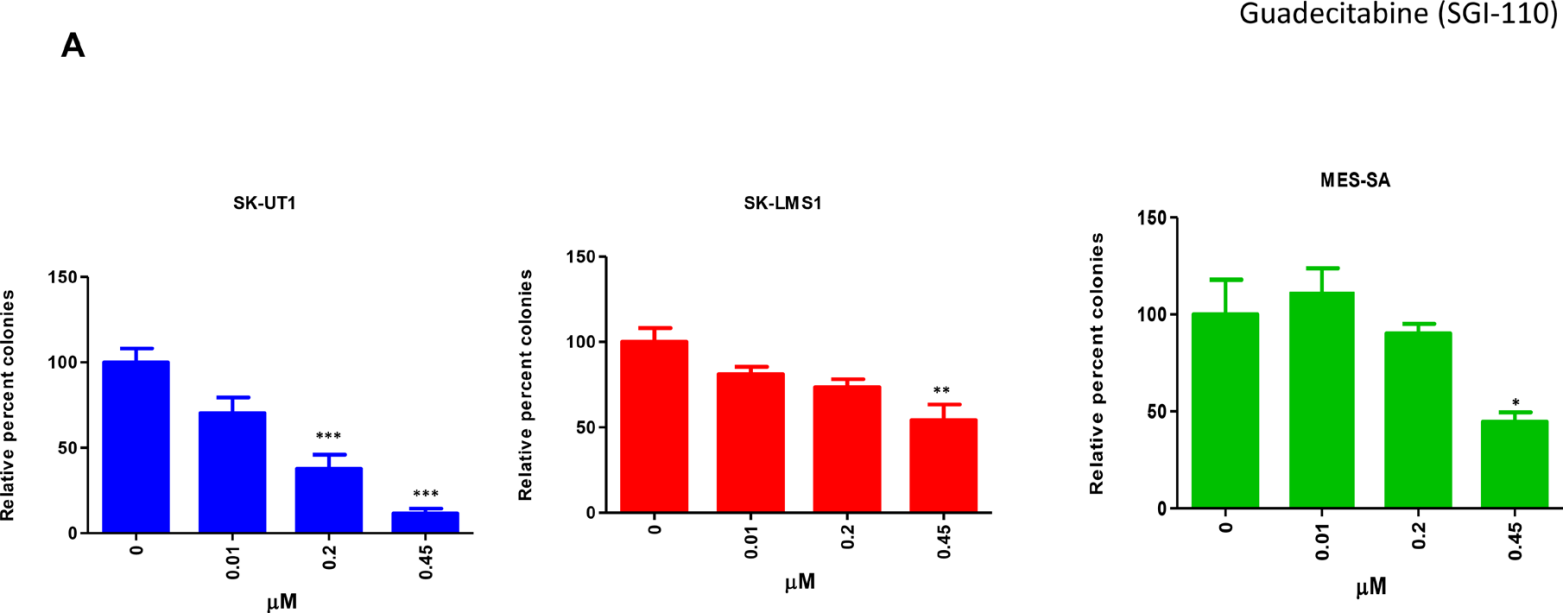

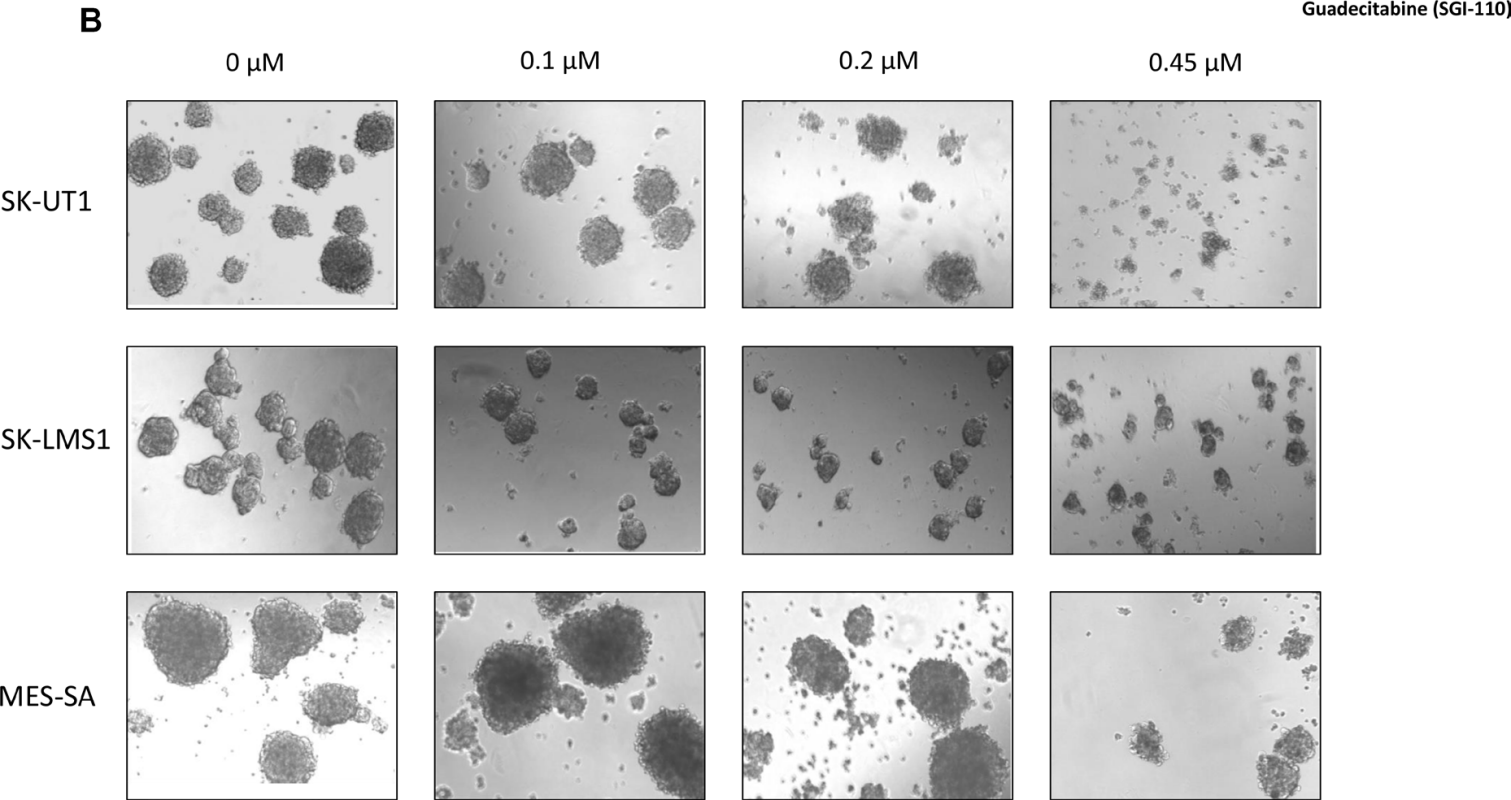

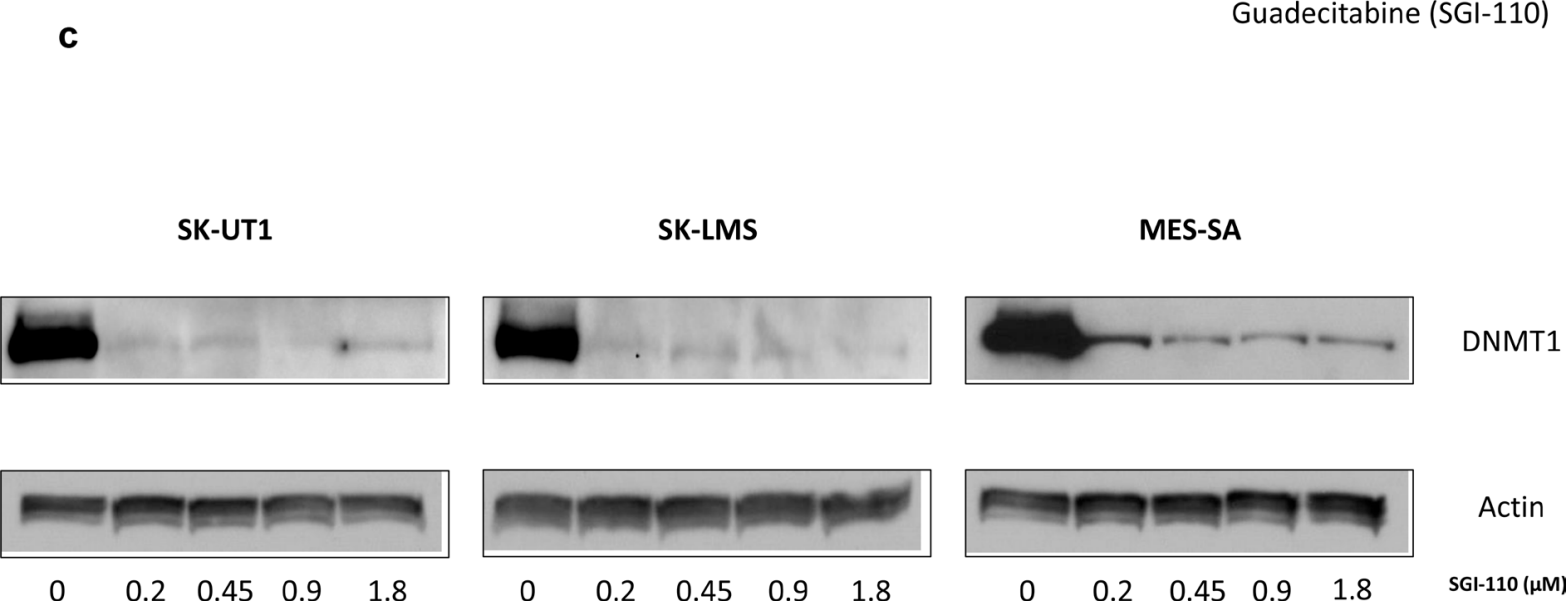
Effect of Guadecitabine on colony formation and DNMT1 expression LMS cells were treated with varying concentrations of guadecitabine for three days. For the colony assay, cells were then seeded into methyl cellulose and the plates were incubated at 37° C at 5% CO_2_ for 7 days after which colonies were manually counted under microscope. The number of colonies were compared relative to control (**A**). Images were taken at 10× magnification using EVOS cell imaging system (**B**). DNMT1 western blots were carried out as mentioned in Methods (**C**). Data shown in colony assay represents mean ± SEM (A).

All three LMS cell lines were treated with serial concentrations of guadecitabine (0.01–0.45 µM) for three days. Colonies were allowed to form over a period of seven days as discussed in materials and methods. In SK-UT1, a significant decrease was observed with the cells exposed to 0.2 µM (*p* < 0.001) as well as to 0.45 µM (*p* < 0.001) with respect to the control (Figure 3A). On the other hand, SK-LMS1 presented a decrease in colony number at 0.45 µM only, relative to control (*p* < 0.01). MES-SA did not show a significant difference in the number of colonies between the control group and any of the treatment groups, except at the 0.45 μM dosage (*p* < 0.05).

Morphologically, the SK-UT1 colonies for the control group were dense, with clearly defined boundaries. As the concentration of guadecitabine increased, there was no change in the size or shape of the colony (Figure 3B), until at the concentration of 0.45 µM, where morphologically the colonies were severely stunted in their growth. For SK-LMS1, while there was no change in the number of colonies across concentrations until the 0.45 μM treatment, physically the colonies appeared smaller in size compared to the control group again; prominently observed at 0.45 µM. MES-SA presented a similar pattern to SK-UT1, with little to no change seen in the size or the morphology of the colonies until a concentration of 0.45 μM was reached. At this level of treatment, colonies became smaller and less compact than at the previous concentrations, and the borders of the colonies were less defined.

### Guadecitabine effectively inhibits DNMT1 protein expression

It has been reported that the primary mechanism of Aza and DAC is the inhibition of DNA Methyltransferase 1 (DNMT1), and it is predicted that guadecitabine will act in a similar fashion to its predecessors. In order to investigate this, all 3 cell lines were treated with guadecitabine for 3 days (0–1.8 μM). After treatment, SK-UT1 and SK-LMS1 showed a complete loss of DNMT1 expression, while MES-SA had a significant reduction compared to the control (Figure 3C).

### The delayed effect of guadecitabine in leiomyosarcoma cell lines amplifies the efficacy of the modulator

#### Delayed impact on proliferation and caspase activity

Previous studies, published by our group, have found that Aza and DAC have a delayed effect, or an anti tumor memory response, when used against haematological and epithelial cancer cells [32].

**Figure 4:**
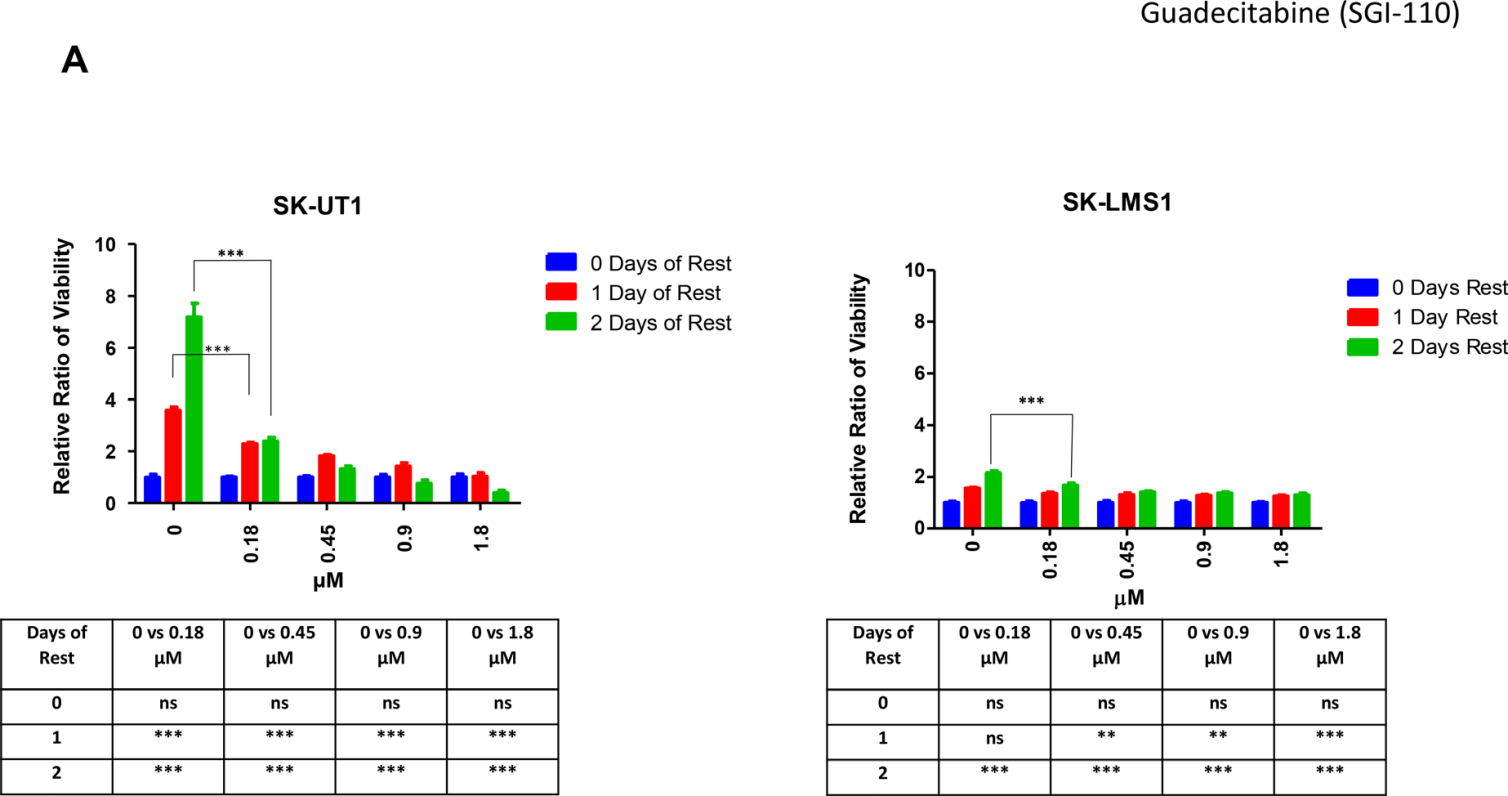

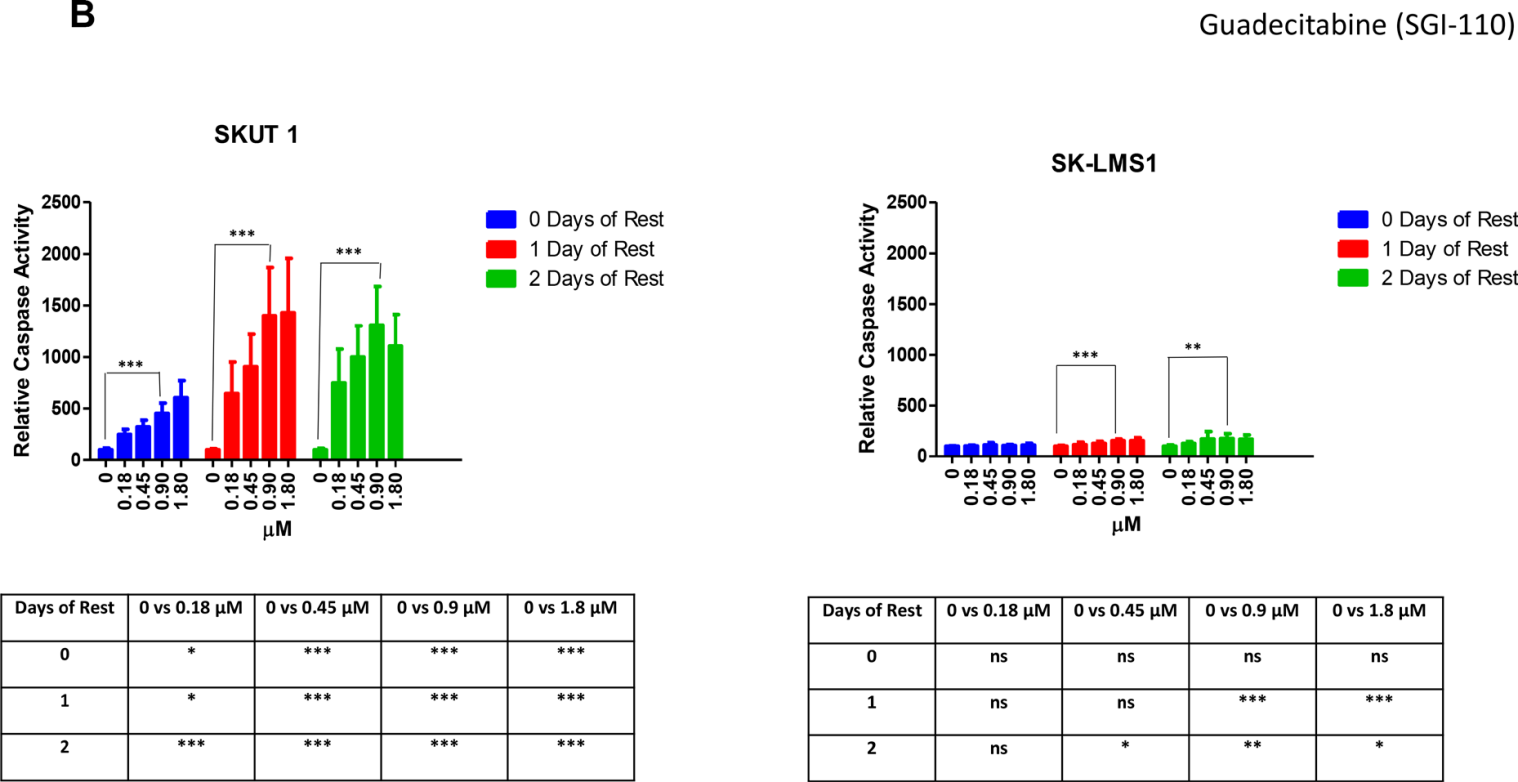

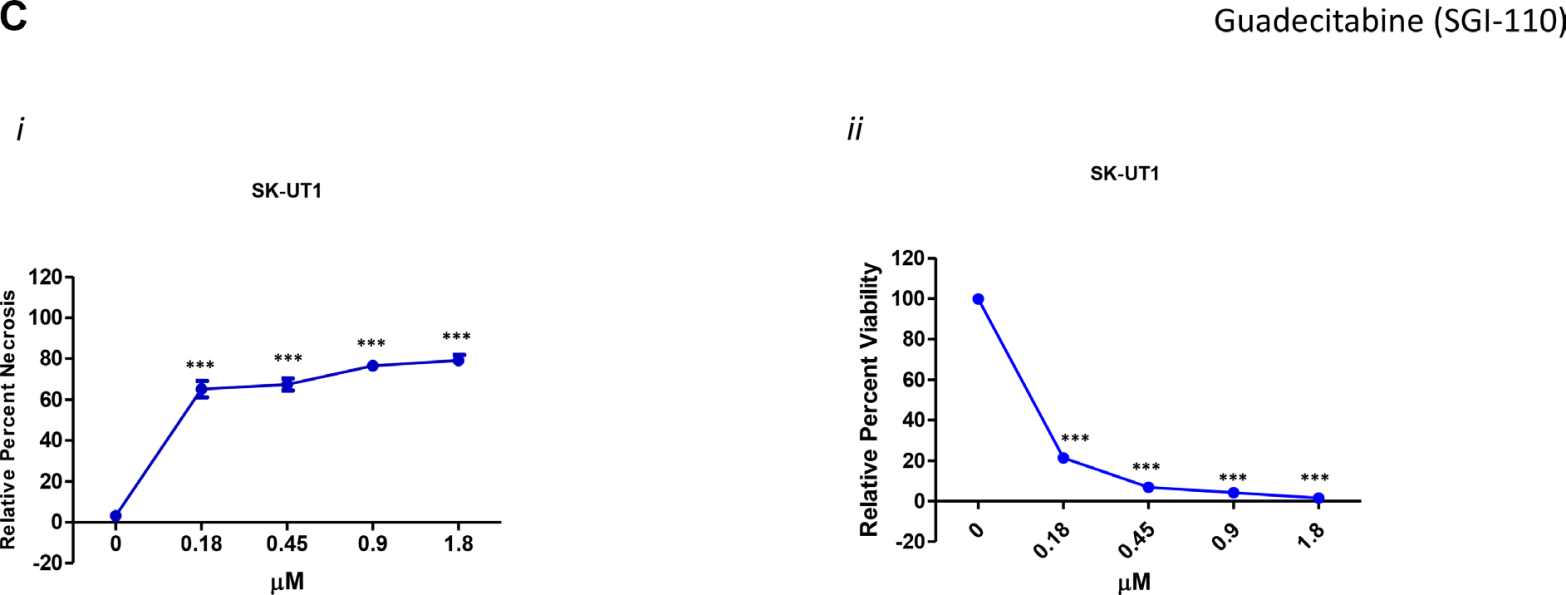

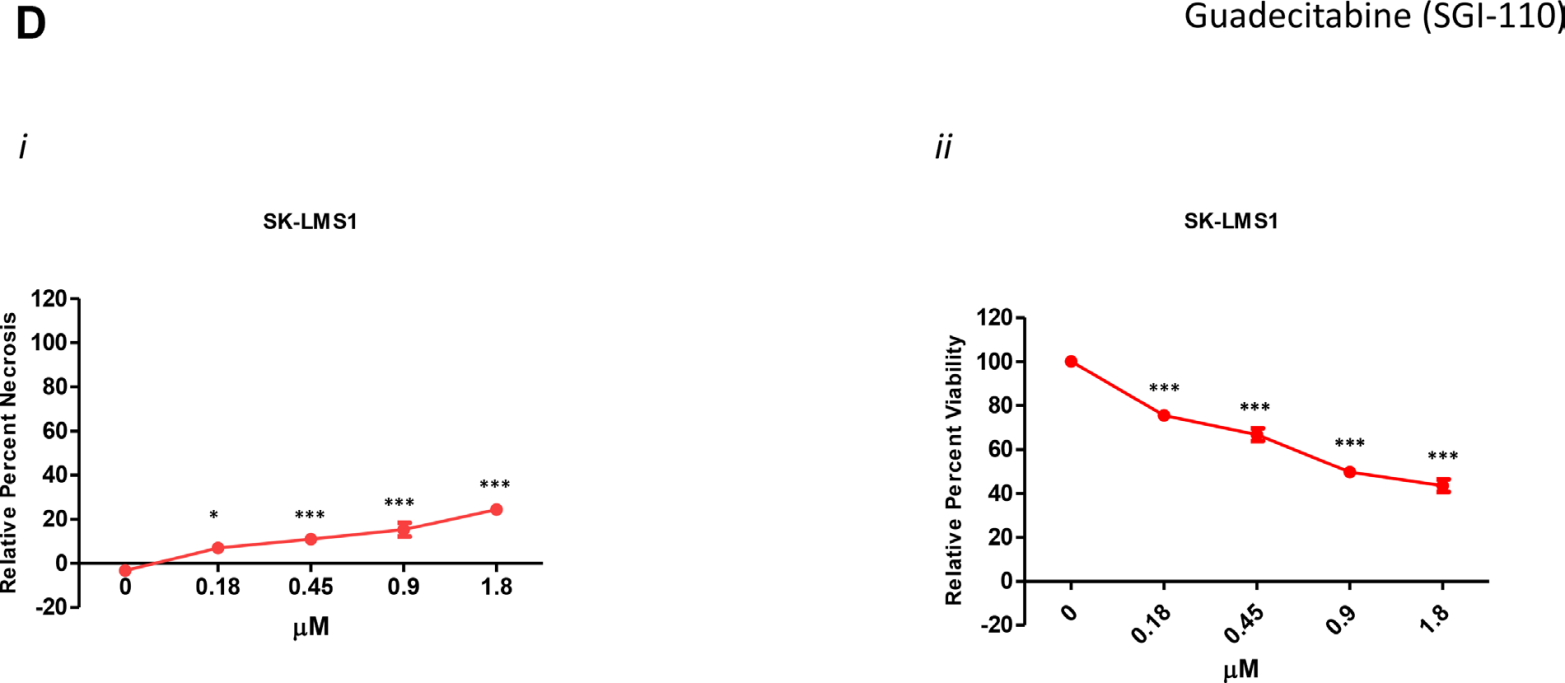

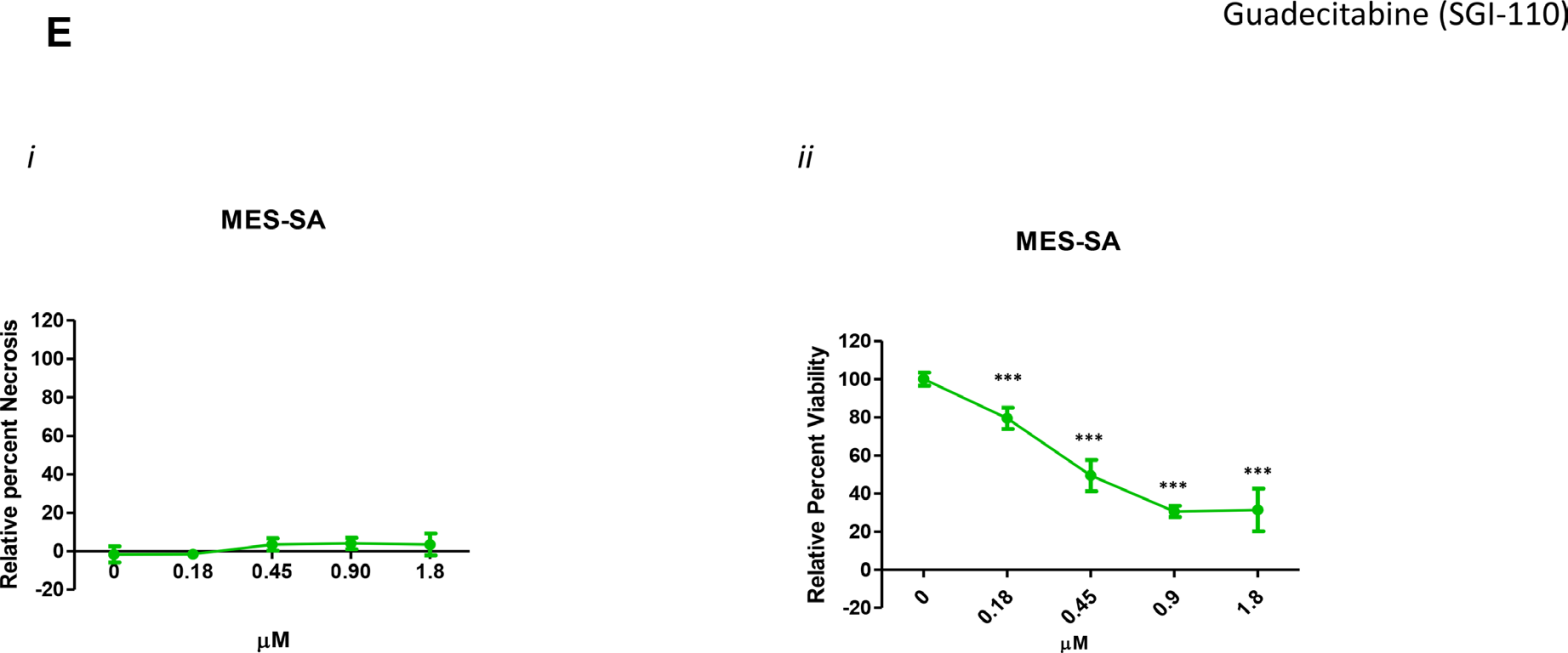

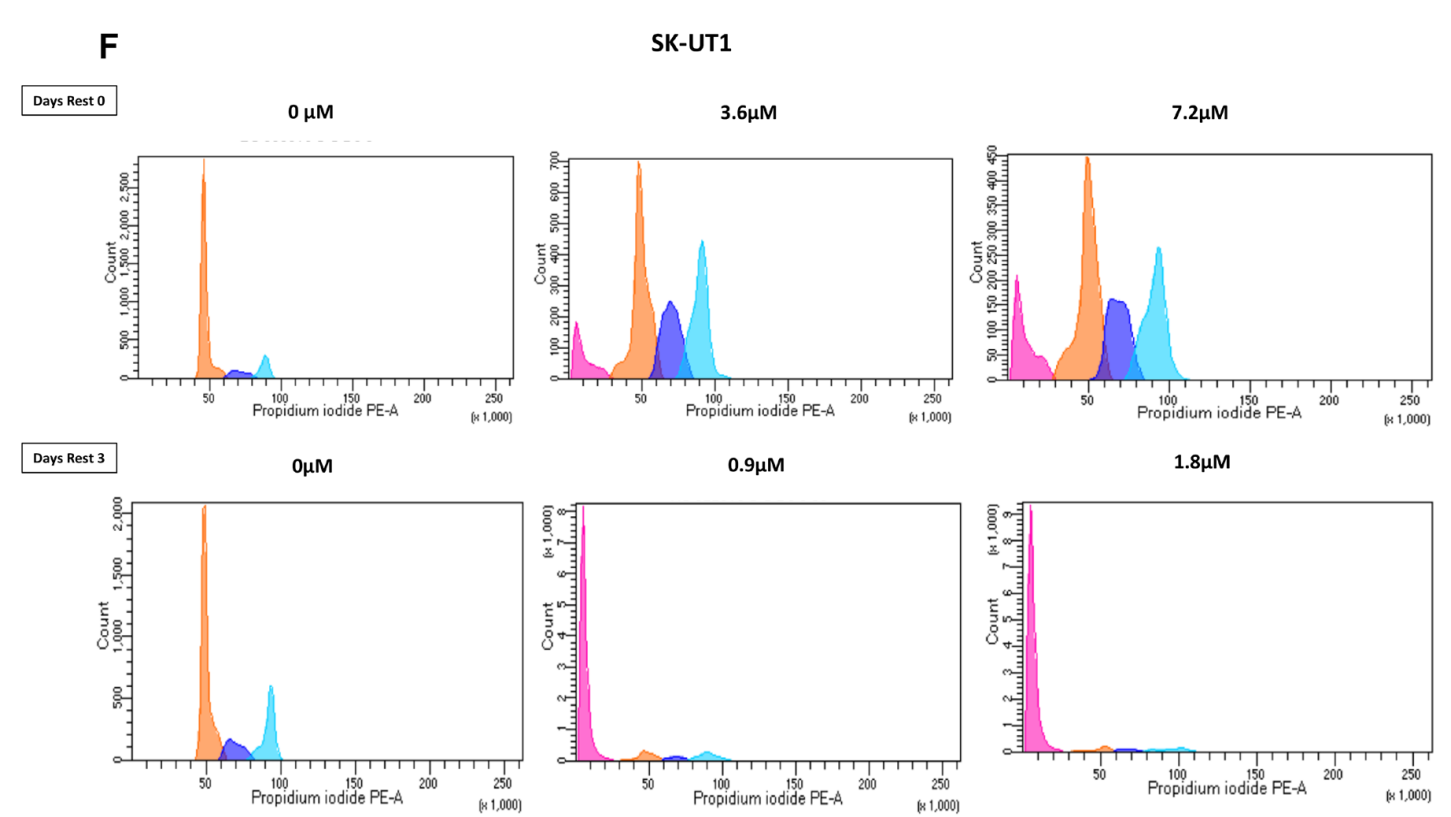

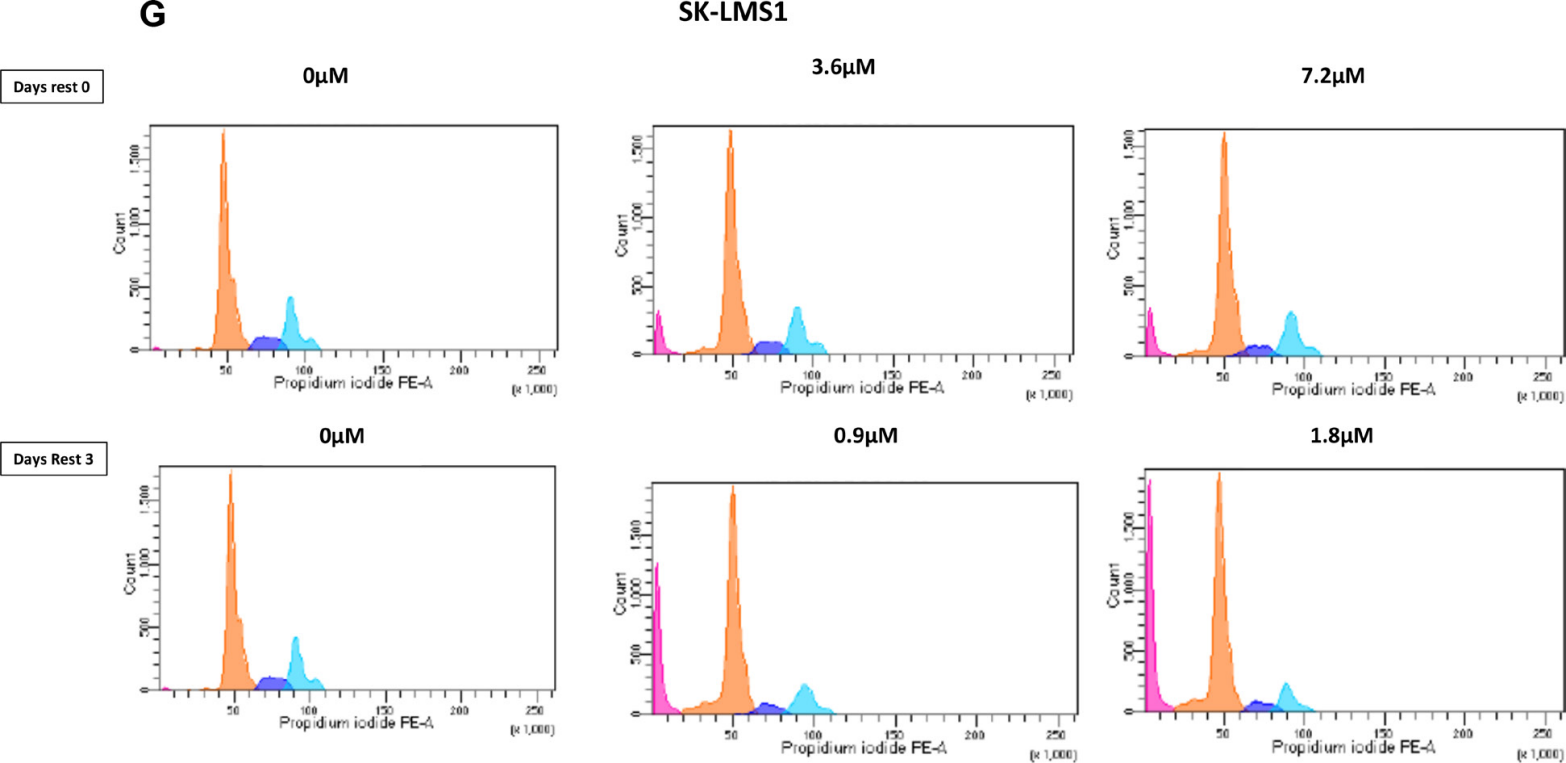
Guadecitabine has a delayed effect on sensitive LMS cell lines SK-UT1 and SK-LMS1 were treated with guadecitabine for 3 days and then allowed to rest for 0–2 days. Following rest, cell survival was measured using a standard MTT assay (**A**), and apoptosis was measured using Caspase 3/7 Glo^®^ (**B**). For both assays, each point was measured as a percentage of the control. Levels of LDH (*i*) (representing necrosis) and cell viability (*ii*) were measured when cells were treated with guadecitabine for three days, followed by three days of rest for SK-UT1 (**C**), SK-LMS1 (**D**), and MES-SA (**E**). SK-UT1 and SK-LMS1 were treated with guadecitabine for 3 days and either fixed immediately or rested for 3 days. Staining was carried out with propidium iodide and run over Flow (**G**–**F**). Data shown represents mean ± SEM, except in Figure 4B. where data is represented as mean ± SD. For Figure G–F, data was analysed using the DIVA software.

This motivated us to investigate whether guadecitabine had similar delayed effects, as it is closely related to DAC. To characterize this delayed effect, only the two extremes of sensitivity from the cell lines tested (SK-UT1 and SK-LMS1) were juxtaposed. These cell lines were treated with either diluent alone, or guadecitabine plus diluent in increasing concentrations over a period of 3 days. Following treatment, cells were allowed to rest in fresh media for up to two days (Figure 4A). Guadecitabine demonstrated a marked anti-proliferative effect in both cell lines following a delay, more so in SK-UT1 than SK-LMS1 (Figure 4A). At 0.18 µM concentration, SK-UT1 growth was significantly inhibited following 1 day and 2 days of rest, when compared to 0 days of rest (*p* < 0.001). In addition, SK-UT1, viability was repressed at 2 days of rest by three fold more than the repression seen with 1 day of rest (for day 1 the relative viability dropped from 4 to 2 and for day 2 from 8 to 2) (Figure 4A). SK-LMS1 continued to proliferate after both 1 and 2 days of rest in the control (*p* < 0.001). However, following 2 days of rest, the level of viability seen at 0.18 µM was significantly lower than that seen for the controls (*p* < 0.001).

Next, we assessed if the delayed anti-proliferative effects were a result of apoptosis (Figure 4B). Apoptosis was measured indirectly by quantifying the levels of Caspase 3 and 7 following treatment of guadecitabine with zero to two days of rest. Caspase 3 and 7 were measured in relation to caspase levels in the control and were expressed as a percentage change. For SK-UT1, caspase 3/7 levels increased dramatically following one and two days of rest when compared to the levels at zero days of rest. At 0.9 μM, caspase 3/7 levels were almost three times higher following 1 and 2 days of rest when compared to no rest (*p* < 0.001). For SK-LMS1, while the increase was on a smaller scale, treatment with 0.9 μM still elicited a significant increase in caspase 3/7 levels following 1 and 2 days (*p* < 0.001 and *p* < 0.01 respectively) of rest compared to no rest.

### Delayed impact on levels of necrosis and viability

lactate dehydrogenase (LDH), which is released upon overall cell death, was used to measure levels of cell death in order to further characterize the effect of a rest period of three days following guadecitabine treatment. In SK-UT1, an increase of LDH was observed in all of the guadecitabine treatment groups (Figure 4C*i*). Specifically, a 60% increase in LDH was observed at 0.18 μM compared to the control (Figure 4C*i*). In parallel, an 80% decrease in cellular growth was seen at the same concentration (Figure 4C*ii*). In SK-LMS1, only an increase of 5% in LDH levels was observed at a concentration of 0.18 μM as compared with the control (Figure 4D*i*). However, a decrease in cell viability of 30% was seen at 0.18 μM following three days of rest (Figure 4D*ii*). MES-SA showed an initial decrease of 20% in viability at 0.18 μM of guadecitabine, a trend that continued as the amount of guadecitabine used increased (Figure 4E*ii*). However, no increase in LDH was observe to parallel the drop in viability (Figure 4E*i*).

Following treatment, all three cell lines showed no increase in necrosis without a rest period (Supplementary Figure 1). This, combined with the above experiments, demonstrate that in the more sensitive cell line SK-UT1, guadecitabine treatment leads to cell death, regardless of rest. In the more resistant line SK-LMS1, moderate death is not observed until the effects of guadecitabine are amplified by a rest period. No death is observed without rest. For MES-SA, no death is observed with or without a rest period.

### Delayed impact on cell cycle

As SK-LMS1 and MES-SA demonstrated decreases in viability with minimal to no death respectively, we next wanted to investigate if guadecitabine arrests cell cycle progression. To accomplish this, we treated our two differentiated cell lines (SK-UT1 and SK-LMS1) with guadecitabine for 3 days, and then cells were stained with propidium iodide and the staining was analyzed over Flow Cytometry.

For SK-UT1 with no rest, treatment with either 3.6 μM and 7.2 μM doubled the percentage of cells arrested in both the S phase (dark blue) and the G2_M phase (light blue) (Figure 4F). Furthermore, while there is relatively no apoptosis observed in the control (G0G1-pink), in both treatment groups apoptosis was observed in around 10% of the total population.

When the SK-UT1 cells were allowed to rest before being fixed, the percentage of cells in the S phase and G2_M phase decreased in our treatment groups (0.9 μM and 1.8 μM). However, the percentage of apoptotic cells increased dramatically from 0.3% of the total population in the control to around 70% for 0.9 μM and around 76% for 1.8 μM. This indicates apoptosis in the more responsive cell line following rest.

For unrested SK-LMS1, treatment with 3.6 μM and 7.2 μM of guadecitabine over 3 days lead to no change in the percentage of cells arrested in the S phase (dark blue) or the G2_M phase (light blue) when compared with the control (Figure 4G). However, the percentage of apoptotic cells (G0G1-pink) increased from 0% to approximately 8% percent of the population for both treatment groups.

When SK-LMS1 was allowed to rest following treatment with guadecitabine at 0.9 μM and 1.8 μM, the percentage of cells arrested in the S phase and G2_M decreased slightly. On the other hand, percentage of apoptotic cells increased from 1% percent in the control to about 25% in both treatment groups. This again confirms that while SK-LMS1 is susceptible to the effects of guadecitabine, it is not as sensitive to the epigenetic modulator as the cell line SK-UT1.

### Leiomyosarcoma cell lines showed decrease in tumorigenesis when treated with Guadecitabine

Given the remarkable *in vitro* anti-proliferative responses seen in LMS cell lines with low doses of epigenetic modulators, we explored if these effects could be seen *in vivo*. Guadecitabine was used for *in vivo* testing since it appeared to have increased potency as compared to Aza and DAC from *in vitro* models.

**Figure 5:**
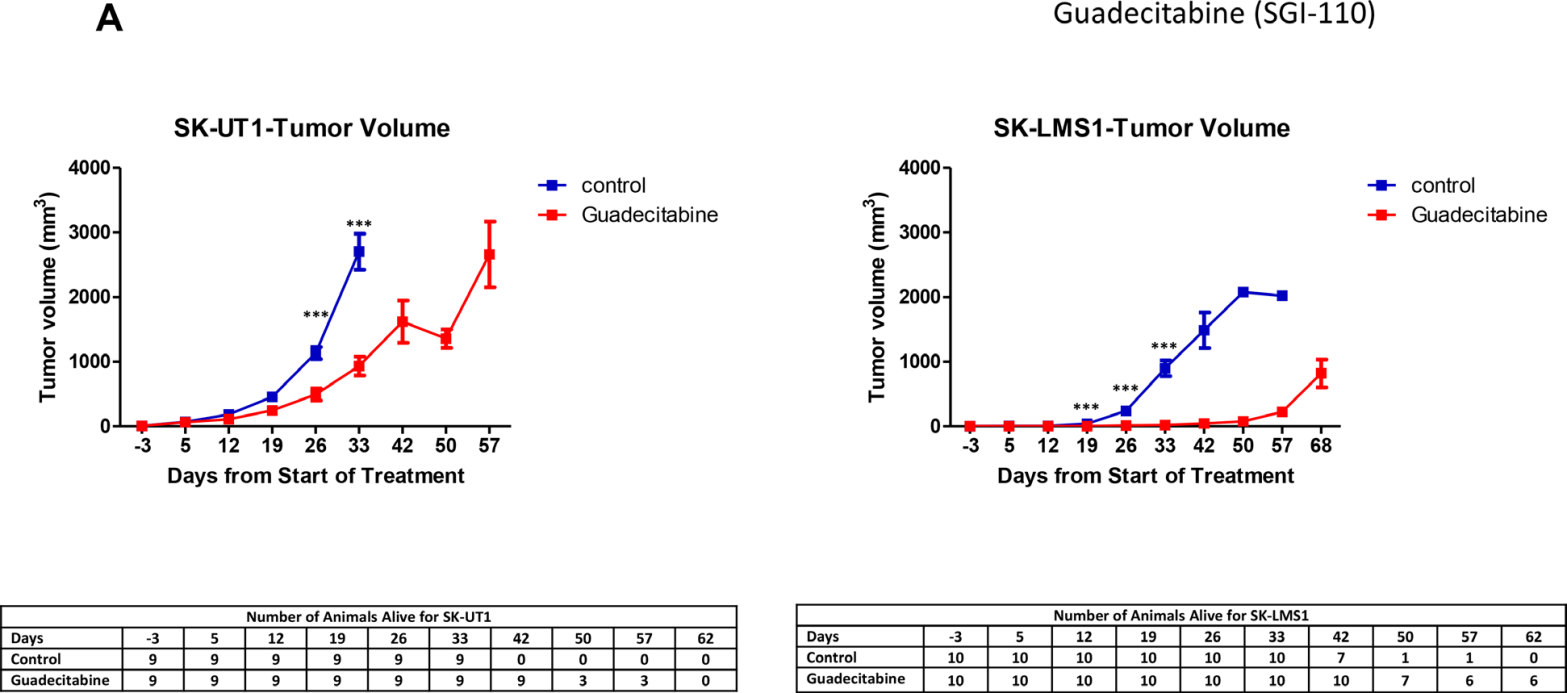

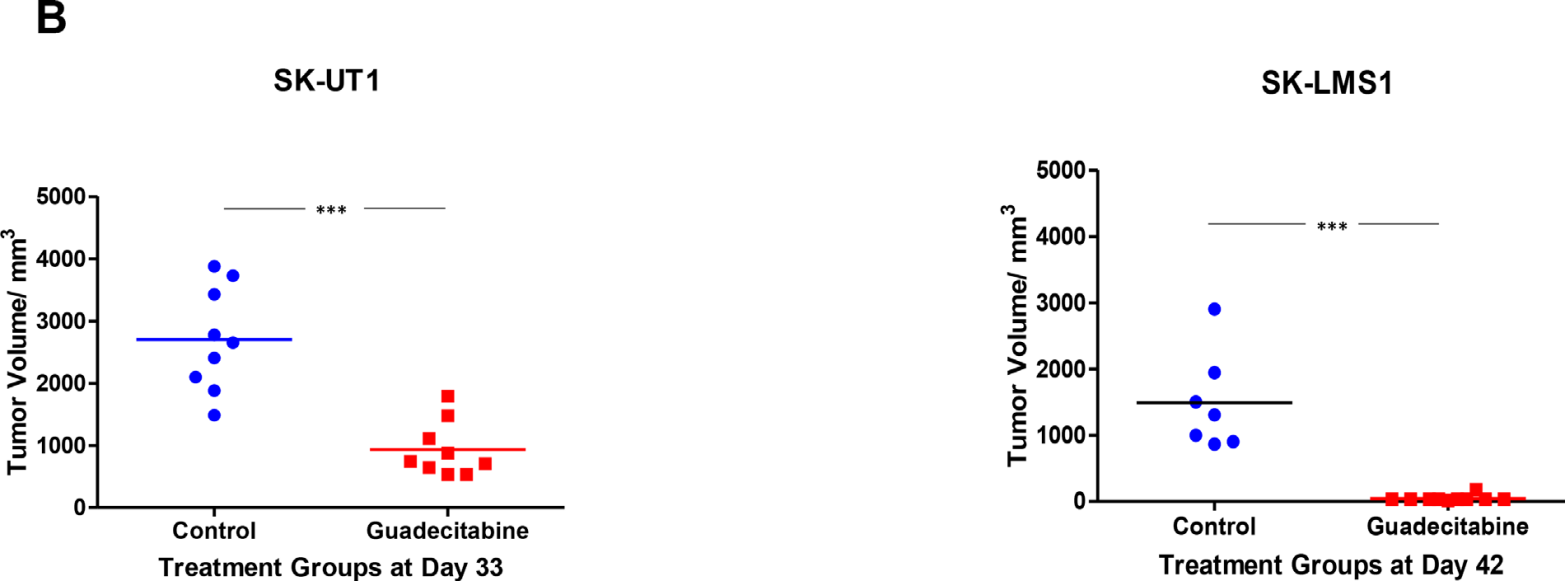

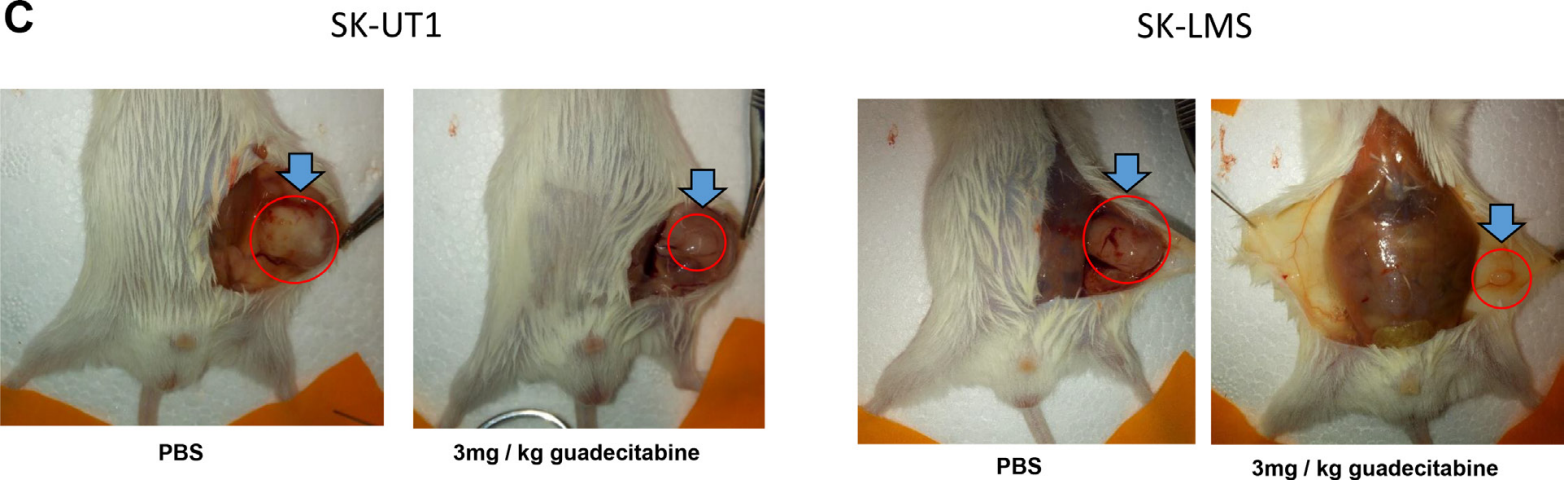

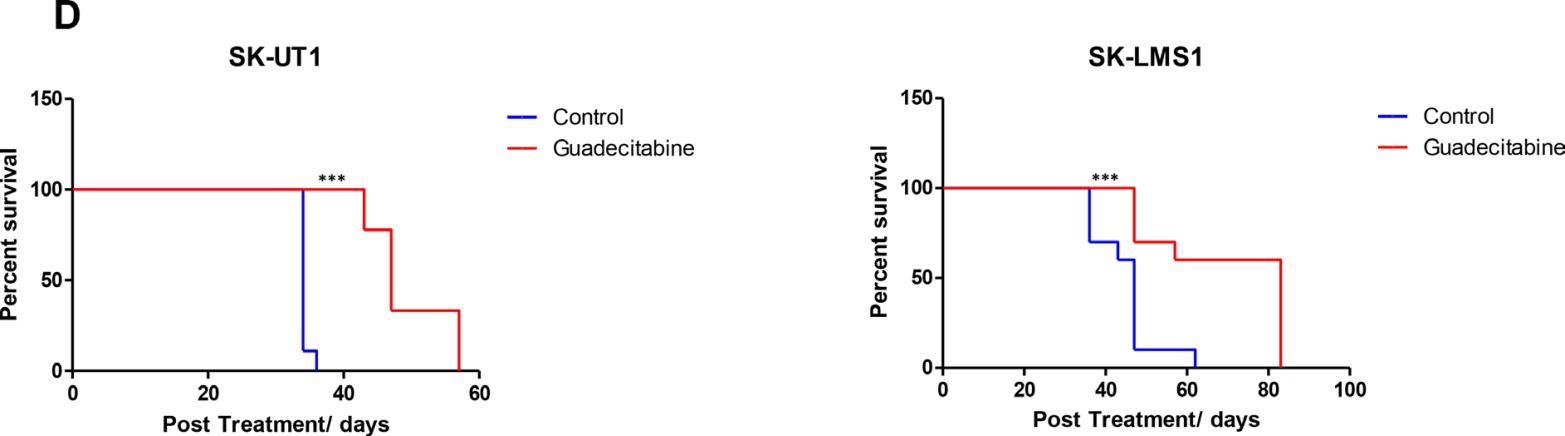

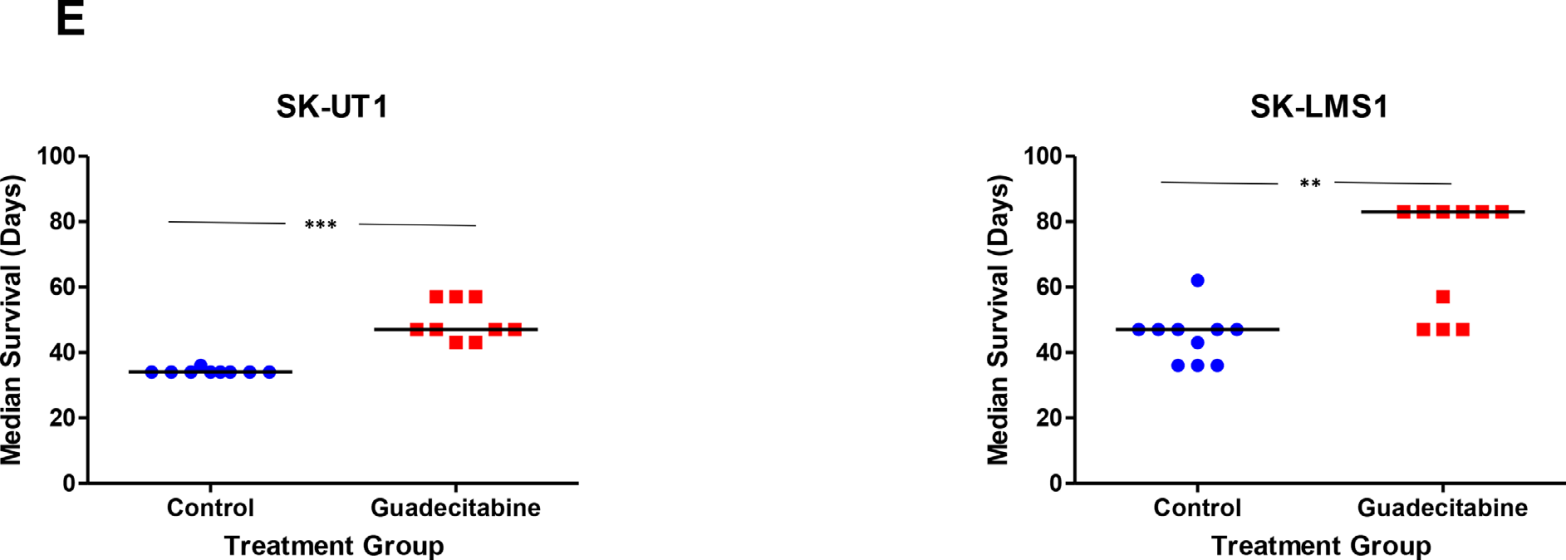
Decrease in tumor volume with Guadecitabine in xenograft model NOD/SCID mice were treated biweekly with 3 mg/kg guadecitabine following growth of palpable tumor xenografts of SK-UT1 and SK-LMS1. The effect of treatment on tumor volume was compared with the control group, both during the course of the study (**A**), as well at the endpoint (**B**). Images of treatment *vs*. control groups for both cell lines are represented at the termination of the study (**C**). Survival time is shown as a Kaplan-Meier Curve demonstrating the effect of biweekly treatment with 3 mg/kg guadecitabine on the survival of NOD/SCID mice with SK-LMS1 and SK-UT1 xenografts (**D**). Median survival time was calculated for treatment vs. control group for both cell lines (**E**). Data shown represents mean ± SEM, except in Figure 5D. which represents individual survival time and Figure 5E. which are median values.

SK-UT1 and SK-LMS1, as our differentiated cell lines (2 × 10^6^ cells per animal), were injected subcutaneously into the right flanks of NOD/SCID mice. Once tumors were palpable (>5 mm^3^), the mice were treated with a bi-weekly regimen of subcutaneous injections of guadecitabine (3 mg/kg) or PBS. Figure 5 demonstrates the effects of guadecitabine therapy on size for both LMS cell line xenograft models. The rate of tumour growth was significantly reduced in those animals treated with guadecitabine *versus* PBS (*p* < 0.001) over a period of 30–60 days for both LMS cell lines (Figure 5A).

Mice transplanted with SK-UT1 and treated with guadecitabine had tumors with less than half the volume when compared to control tumors at the pre-designated time point for sacrifice of controls (SK-UT1: 33 days; SK-LMS1: 42 days) (Figure 5A). For SK-UT1 control mice, the average tumor volume was just under 3000 mm^3^, while the guadecitabine treated group had an average tumor volume of around 1000 mm^3^ (*p* < 0.001) 33 days following the beginning of treatment. For mice inoculated with SK-LMS1 cells, the growth of guadecitabine treated tumors was severely delayed, while the tumors in controls grew rapidly. For control mice, the average tumor volume at the experimental end was around 1500 mm^3^, while the guadecitabine treated group had an average of just above 10 mm^3^ (*p* < 0.001) (Figure 5B). For the mice in the guadecitabine treated SK-LMS1 cohort, tumors were allowed to continue to grow until they reached an average volume of around 1000 mm^3^. This was done in order to confirm the presence of a viable tumor.

Morphology of the tumors was assessed after the animals were sacrificed. As shown by the images, guadecitabine treated tumors were smaller in size when compared to their control counterparts for animals inoculated with either SK-UT1 or SK-LMS1 (Figure 5C).

In addition to inhibiting tumor growth, guadecitabine treatment led to an increase in the duration of survival in the experimental groups (in both cell lines) compared to controls (Figure 5D). For mice harbouring SK-UT1, animals treated with guadecitabine survived longer than the control group (median survival: 34 days control vs. 47 days guadecitabine) (*p* < 0.001) (Figure 5E). For the SK-LMS1 mice, the treatment group also survived significantly longer than the control, (median survival 47 days control vs. 83 days guadecitabine) (*p* < 0.001). During this interval, mice from all four groups were seen to gain weight (data not shown).

## DISCUSSION

The prognosis for sarcoma patients remains very poor, in spite of progress that has been made in other types of cancer [33, 34]. The most common method of treating this disease is through surgical resection. The systemic chemotherapy options available include the drugs docetaxel and gemcitabine which have a complete response in only 5% of the patients [35, 36]. Other chemotherapy drugs like methotrexate, doxorubicin, and cisplatin (MAP) have been evaluated in clinical trials with little success to date [37].

Numerous clinical trials have targeted aberrant receptor expression, such as vascular endothelial growth factor receptors (VEGFR), estrogen receptors (ER), and progesterone receptors (PR) [38–42]. None of these trials has had a significant effect on disease progression or survival of patients. Other agents such as rapamycin, a mTOR inhibitor, and Topotecan, a topoisomerase I inhibitor, have been found to have either limited or conflicting results in various clinical trials [43–45]. Therefore, it should be of great urgency to investigate and develop new targeted therapies to try and alter the rapid, often terminal progression of this disease.

To date, very little research has been done with the characterization of sarcoma epigenetics. As elevated expression of histone deacetylase (HDAC) has been observed in stromal sarcomas, newer studies investigated the efficacy of HDAC inhibitors both *in vitro* and in the clinic [46–48]. Further research is required to determine the true effectiveness of these HDAC inhibitors. In spite of the attempts to understand histone acetylation, no studies have yet investigated the effects of DNA methylation.

Guadecitabine, of the three DNMTi, was the most effective at slowing cellular viability in the cell line with the highest responsivity (SK-UT1), and the only drug to accomplish this at a clinically relevant concentration. These results were replicated in a 3D model via a colony formation assay, where guadecitabine decreased the number of colonies in the more sensitive cell line (variation in cell line sensitivity is most likely due to overall methylation status of the cell lines [8]).

Our group has shown that Aza and DAC have a delayed effect when treating hematological tumors [32]. A similar memory effect was seen for all three cell lines in terms of viability following guadecitabine treatment. However, it would seem that this amplified decrease was not achieved in the same manner. Following a rest period, both SK-UT1 and SK-LMS1 had significant and moderate (respectively) increases in cytotoxicity, implicating death in the decreased cell counts. MES-SA, did not demonstrate any cytotoxicity following both 0 and 3 days of rest to explain the diminished viability seen in the MTT and colony assays. This would imply that while this modulator is diminishing the cell viability even in the more resistant cell lines, it must be accomplishing this through some other mechanism rather than death.

Caspase 3/7 expression, as an indicator of potential apoptotic death, was also amplified following a rest period in SK-UT1 and SK-LMS1. In SK-UT1, the effect was drastic, while in SK-LMS1 the increase was not as profound. MES-SA, on the other hand, did not exhibit a noticeable increase in Caspase activity (Supplementary Figure 2). This is in accordance with our above theory, that while guadecitabine is decreasing viability, it can achieve this outside of a cellular death pathway.

Aza has been previously described to arrest cell cycles in the G2 phase [49]. Since we have observed decreases in viability not due to cell death (i.e. our MES-SA cell line), we wanted to investigate if guadacitabine acted in a similar effect to its analogue. In our cell cycle experiment, the SK-UT1 populations treated with guadecitabine, and unrested, demonstrated large percentages of the cells arrested in the S and G2_M phase when compared to the control. This shows that guadecitabine acts in a similar manner to its epigenetic modulating predecessor.

Both LMS cell lines demonstrated a significant response to treatment of guadecitabine when moved into an *in vivo* model. Tumor growth was severely stunted for each cell line when compared to the control, in both the more and less resistant cell line. Guadecitabine did allow a large percentage of the treatment groups for both cell lines to survive longer than they would have if they had not received treatment (i.e., the control group).

Our results show that the hypomethylating agent guadecitabine appears to have greater efficacy in treatment of LMS cell lines *in vitro* than its counterparts. These anti-tumor effects were also replicated with *in vivo* models. Further studies are needed to elucidate the mechanism of this finding.

## MATERIALS AND METHODS

### Reagents

The following leiomyosarcoma cell lines were purchased from the ATCC: SK-LMS1 (ATCC^®^ HTB-88^™^, leiomyosarcoma of the vulva), SK-UT1 (ATCC^®^ HTB114^™^, uterine leiomyosarcoma), and MES-SA (ATCC^®^ CRL-1976^™^, poorly differentiated uterine sarcoma). CellTox^™^ Green Cytotoxicity Assay (cat. No. G8741), CellTiter96 Cell Proliferation Assay (cat. No. G3580) and Caspase Glo^®^ Assay (cat. No. G8090) were purchased from Promega. 5-azacitidine (cat. No. A2385) and 5-aza-2′-deoxycytidine (cat. No. A3656) were purchased from Sigma-Aldrich. Guadecitabine (SGI 110) was supplied by ASTEX pharmaceuticals. NOD/SCID mice were purchased from (Jackson Laboratories).

### Cell culture

SK-LMS1 and SK-UT1 cell lines were cultured in Minimum Essential Media (MEM) (cat. No. 10-010 CV) with 10% Fetal Bovine Serum (cat.No.100-106) MES-SA cells were grown in McCoys media (cat. No. 10-050 CV) containing 10% FBS.

### Cell viability assays

An MTT Assay was used as a measure of cell viability. For each cell line, either 1000 (SK-UT1) or 1500 (SK-LMS1) cells per well were seeded into a 96 well plate and allowed to adhere overnight. Treatment with 5-azacitidine (Aza) (0.1–5 μM), 5-aza-2-deoxycytidine (DAC) (0.1–5 μM), or guadecitabine (SGI-110) (0.02–9.0 μM) was performed in triplicate. Treatment with various epigenetic modulators was compared with controls, which were cells cultured in basal media containing an equivalent amount of the drug’s solvent. Media was renewed daily, and the cells were incubated for a maximum of five days. At the indicated time points, the appropriate amount of MTT-reagent, as per manufacturer’s recommendation (Promega), was added to the media and incubated at 37° C, 5% CO_2_ for two to four hours. The absorbance was measured at a wavelength of 490 nm spectrophotometric ally (Biorad iMark^™^ Microplate Reader). Each data point was calculated relative to control and expressed in terms of percent survival and plotted using Graph Pad Prism^©^. A minimum of three experimental replicates were conducted.

MTT-assays were also used to test whether LMS cell lines showed a delayed response to treatment with increasing concentrations of the DNMTi guadecitabine. Cells were seeded into a T75 flask and allowed to adhere overnight. Treatment with guadecitabine (0.18–1.80 μM) was performed over a total of 3 days. Control cells were treated with an equivalent volume of diluent. All media was renewed daily. Following treatment, cells were plated in 96 well plates and incubated in drug-free media for 1–3 days. Absorbance was measured as described above. A minimum of three experiments were conducted to confirm results.

### Caspase 3/7 assay

Caspase Glo^®^ Assay kit (Promega) was utilized in order to measure apoptosis by proxy of caspase 3 and 7 activity. This was carried out in order to further characterize the mechanism of DNMTi drug induced apoptosis. Cells were cultured in the above-mentioned conditions, and the luminescent signal was assessed using the manufacturer’s instructions. The readout for each drug concentration was determined as a relative percentage to the control ((experimental/control) ^*^100) and plotted using Graph Pad Prism.

Furthermore, levels of apoptosis for the guadecitabine treated LMS cells were quantified during the delayed response experiment using the abovementioned Promega Caspase 3/7 kit. Following treatment, cell lines were reseeded in drug free media in 96 well plates. The plates were incubated in the same conditions as the MTT delayed response experiment for 1, 2, or 3 days. Luminescence was measured as previously described above.

### Colony formation assay

All three cell lines were cultured as a monolayer for 24 hours prior to treatment. Cells were incubated with different concentrations of guadecitabine for a total period of 3 days. The cells were trypsinized and suspended as a single-cell solution in a methylcellulose-medium containing 3% methylcellulose, culture media, 10% BSA, fetal bovine serum, L-glutamine, penicillin/streptomycin and Beta mercapto ethanol [50]. Cells were then aliquoted as duplicate samples at 4 × 10^6^ cells per well. Colonies were allowed to form by incubating plates for 7 days at the above mentioned conditions, then assessed for morphology and counted manually with microscopy (at 10× magnification). Colonies were defined as distinct groups of fifty or more cells. Pictures were taken using the EVOS Cell Imaging System (Thermo-Fisher Scientific).

### Western blotting

All three cell lines were plated into T75 flasks and treated with guadecitabine for 3 days (0–1.8 μM). Westerns were carried out as previously detailed [51].

### Necrosis assay

LMS cell lines were plated into a 96 well format and treated with either Aza or guadecitabine for three days. Each day, the media in each well was collected and stored at 4° C. Following treatment, lactate dehydrogenase (LDH) activity was measured from the collected media using a Thermo Scientific Cytotoxicity Kit (cat. No. 88954).

Similarly, to measure delayed cytotoxicity following treatment of guadecitabine, LMS cells were again seeded into a 96 well plate format and treated with guadecitabine for three days. Following the third day of treatment, the cells were allowed to rest for three days in fresh media (rest was defined as a period in which cells were not exposed to drugs and were maintained in drug-free media). On the third day of rest, the media was collected from plates and again LDH activity was measured used the same Thermo Scientific Cytotoxicity Kit. The data was analysed according to manufacturer’s instructions.

### Flow cytometry

SK-UT1 and SK-LMS1 cells were plated into a T75 flask and treated with varying concentrations of guadecitabine for 3 days. Media was collected each day and floating cells were pelleted. Following treatment, the remaining cells were either immediately fixed in chilled 70% ethanol and stored at –20° C, or allowed to rest in fresh media for an additional 3 days before they were fixed. Cells were stained and data was acquired as described previously [52]. The data was analysed using DIVA software.

### Animal experiments

SK-LMS1 and SK-UT1 cell lines were selected for further study of guadecitabine in an *in vivo* xenograft model. All animal studies were conducted in adherence with Johns Hopkins protocols for animal care and use.

Two million SK-UT1 and SK-LMS1 cells were suspended in 200 µL 1:1 Matrigel: PBS and injected in the right flank of NOD/SCID mice. Four experimental groups were defined for each cell line, each containing 10 mice per group: Group A - SK-UT1, biweekly (defined as twice a week) treatment with PBS; Group B: SK-UT1, biweekly treatment with 3 mg/kg guadecitabine; Group C: SK-LMS1, biweekly treatment with PBS; Group D: SK-LMS1, biweekly treatment with 3 mg/kg guadecitabine. Treatment was initiated (considered Day 0) upon identification of a palpable tumor. Tumor size was recorded biweekly, whereas body weight was registered weekly. Behaviour, as a measure of animal health and discomfort, was also observed twice a week. Tumor volume was calculated using the equation (Volume = ((Width^2^)^*^Length)/2) [53]. Mice were sacrificed once the tumor burden pasted the acceptable limits established in our animal protocol (above 2 cm tumor size).

### Statistical analysis

The IC_50_ values for the *in vitro* cell viability assays were determined using a standard slope equation Y = mX + C by fitting the line to a linear regression using Microsoft Excel. The calculated IC_50_ values were plotted using Graph Pad Prism 6. Using the same software, statistically significant differences within treatment groups across time points were determined using one-way ANOVA with Tukey’s Post hoc and non-parametric *t*-tests. Significance was considered at either (^**^p < 0.01) or (^***^p < 0.001) depending on the assay.

## SUPPLEMENTARY MATERIALS FIGURES



## Abbreviations

LMS: leiomyosarcoma
Aza: 5-azacitidine
DAC: Decitabine
SGI-110: Guadecitabine
