# Supplementary material for: Treatment with epigenetic agents profoundly inhibits tumor growth in leiomyosarcoma
Source: Oncotarget. 2018 Apr 10;9(27):19379–95. doi: 10.18632/oncotarget.25056 (PMC5922404; doi:10.18632/oncotarget.25056)
Supplement: Supplementary file 1 [file oncotarget-09-19379-s001.pdf]

Treatment with epigenetic agents profoundly inhibits tumor growth in leiomyosarcoma

SUPPLEMENTARY MATERIALS

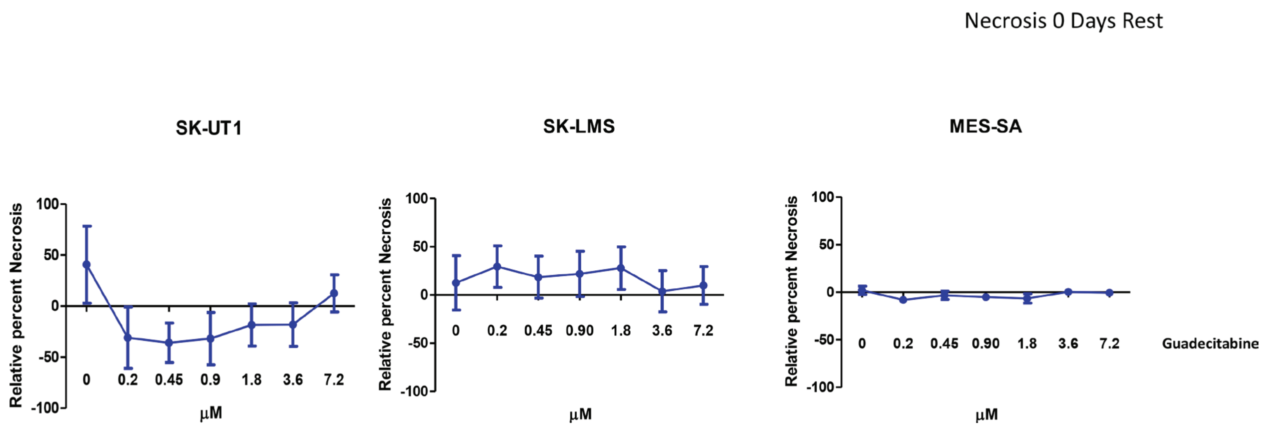

Supplementary Figure 1: Epigenetic modulators have negligible necrosis effect in LMS cell lines. Following 3 days of treatment with guadecitabine, levels of lactate dehydrogenase (LDH) were measured in all 3 cell lines using a cytotoxicity assay. Data points were calculated according to the manufacturer's instructions. Data shown represents mean ± SEM.

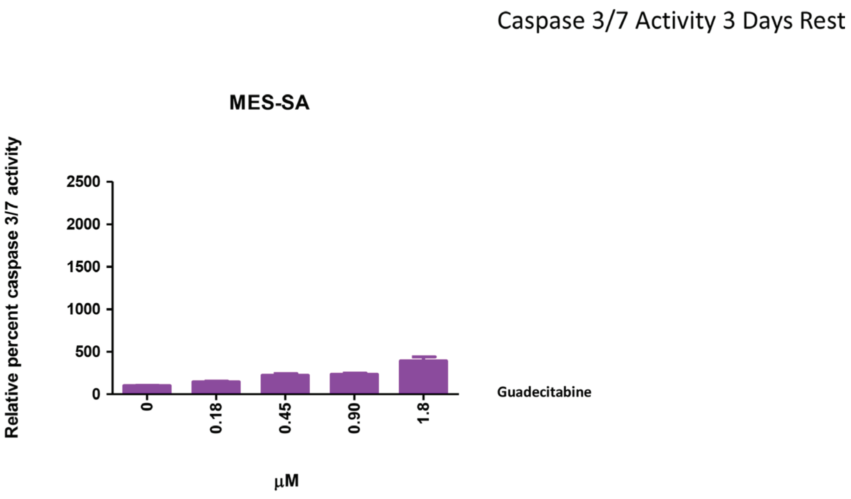

Days of Rest	0 vs 0.18 μM	0 vs 0.45 μM	0 vs 0.9 μM	0 vs 1.8 μM
3	***	***	***	***

Supplementary Figure 2: Delayed Effect on MES-SA caspase levels from guadecitabine. MES-SA cells were treated with guadecitabine for 3 days, and then rested for 3 days. After the rest period, Caspase 3/7 levels were measured using the Promega kit from Methods. Data represents mean ± SEM.
